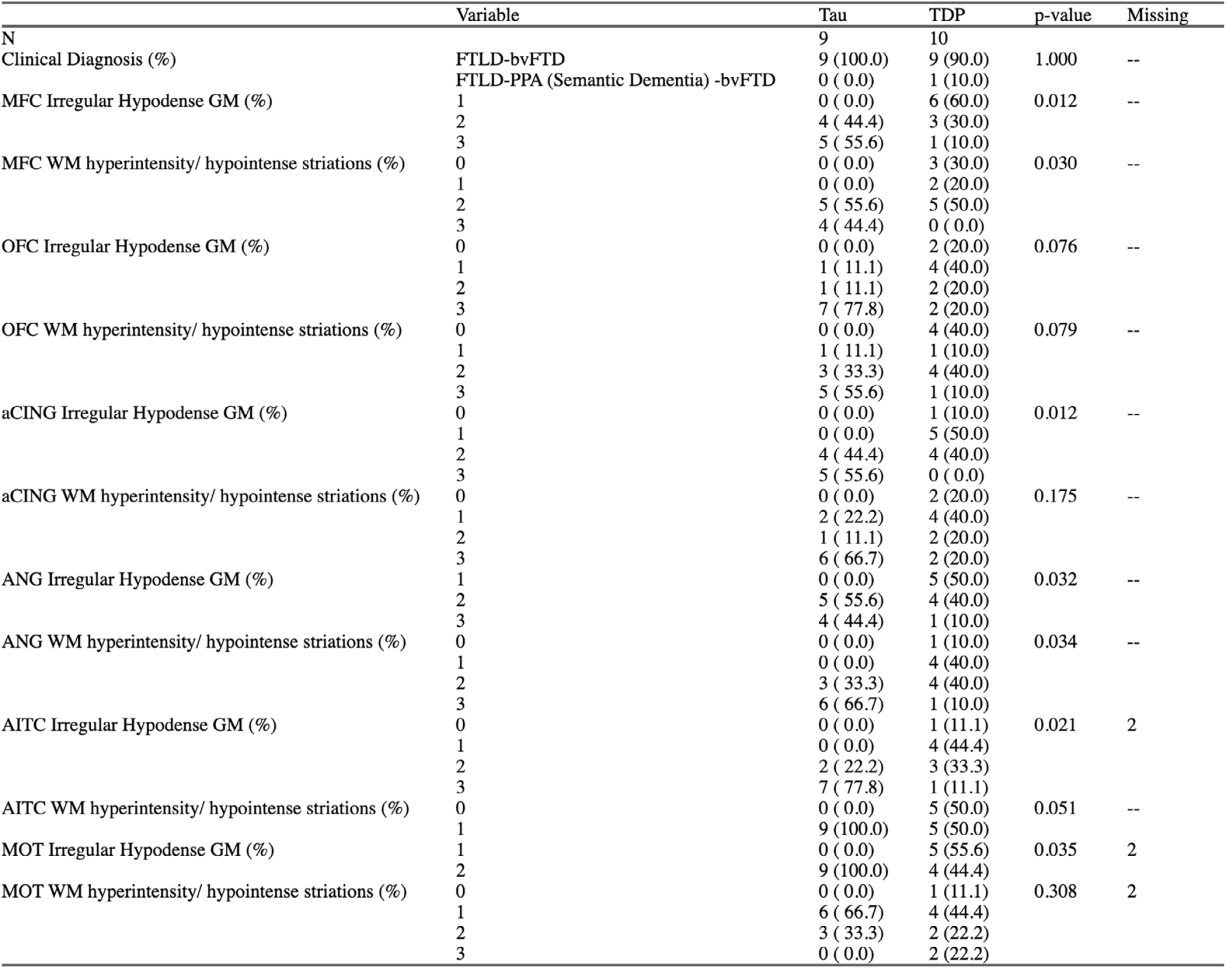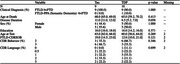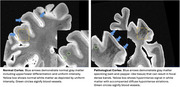# Ultra‐high resolution 7T T2*w *ex vivo* MRI patterns differentiate underlying FTLD pathology in behavioral variant FTD

**DOI:** 10.1002/alz70861_108813

**Published:** 2025-12-23

**Authors:** Sheina Emrani, Eric Teunissen‐Bermeo, Winifred Trotman, Alejandra Bahena, Noah Capp, Daniel T Ohm, Sandhitsu R. Das, David A. Wolk, Eddie B. Lee, Corey T. McMillan, James C. Gee, Paul A. Yushkevich, M. Dylan Tisdall, David J. Irwin

**Affiliations:** ^1^ Department of Neurology, Perelman School of Medicine, University of Pennsylvania, Philadelphia, PA USA; ^2^ University of Pennsylvania, Philadelphia, PA USA; ^3^ Penn Image Computing and Science Laboratory (PICSL), University of Pennsylvania, Philadelphia, PA USA; ^4^ Department of Pathology & Laboratory Medicine, Perelman School of Medicine, University of Pennsylvania, Philadelphia, PA USA; ^5^ Penn Image Computing and Science Laboratory, Philadelphia, PA USA; ^6^ Penn Alzheimer’s Disease Research Center, University of Pennsylvania, Philadelphia, PA USA

## Abstract

**Background:**

Presently, there are no available biomarkers that accurately differentiate behavioral variant frontotemporal dementia (bvFTD) due to heterogenous underlying frontotemporal lobar degeneration (FTLD) Tau (FTLD‐Tau) versus TDP‐43 (FTLD‐TDP) pathology. We developed and utilized ultra‐high resolution (160 µm^3^) *ex vivo* whole‐hemisphere T2‐star(*)‐weighted(w) magnetic resonance imaging (MRI), sensitive to iron in healthy myelin and pathological gliosis, to determine whether patterns of iron‐rich gliosis and laminar degeneration can differentiate autopsy‐confirmed FTLD‐TDP versus FTLD‐Tau in individuals with bvFTD.

**Methods:**

19 patients with bvFTD were included (FTLD‐Tau = 9 (Pick); FTLD‐TDP = 10 (TDP‐A = 5; TDP‐B = 4; TDP‐C = 1; C9orf72 = 4, GRN = 1). Ordinal ratings (0‐3; none‐mild‐moderate‐severe) in the coronal plane were performed, blinded to pathologic diagnosis for two main T2*w contrasts previously observed in FTLD‐Tau (Tisdall et al 2021; Ohm et al 2023): 1) irregular hypodense neuropil of the gray matter (GM) corresponding to iron‐rich gliosis and 2) co‐localized features of hyperintensity from myelin loss and hypointense striations due to iron‐rich gliosis of adjacent deep white matter (WM). Regions included those implicated in both early and later stages of regional pathology in bvFTD (midfrontal cortex [MFC], orbitofrontal cortex [OFC], anterior cingulate [aCING], anterior‐inferior temporal cortex [AITC], angular gyrus [ANG], and motor cortex [MOT]). Kruskal‐Wallis test compared ratings between groups.

**Results:**

There were no statistical differences in age at death, sex, education, or clinical dementia rating score with FTD NACC nearest death (p > 0.05) between group. Irregular hypodense GM in the MOT, MFC, aCING, ANG, AITC and adjacent WM hyperintensity/hypointense striations in the MFC and ANG were statistically greater in FTLD‐Tau vs FTLD‐TDP groups (ps < 0.036), with a trend in WM pathology in the AITC (p = 0.051).

**Conclusions:**

Pathologic subtypes of bvFTD may have partially distinct cellular, laminar and regional patterns of pathology that can inform diagnosis and mechanisms of FTLD proteinopathies. Ultra‐high resolution T2*w MRI with sufficient resolution to detect these differences in vivo could increase diagnostic accuracy of underlying pathology in bvFTD. Future quantitative work with histology validation will elucidate whole‐hemisphere patterns of FTLD cellular pathology and facilitate *in vivo* MRI biomarker development.